# Protocol for assessing neighborhood physical disorder using the YOLOv8 deep learning model

**DOI:** 10.1016/j.xpro.2023.102778

**Published:** 2023-12-15

**Authors:** Yan Li, Yue Ma, Ying Long

**Affiliations:** 1 School of Architecture, Tsinghua University, Beijing 100084, China; 2 School of Architecture and Hang Lung Center for Real Estate, Key Laboratory of Eco Planning & Green Building, Ministry of Education, Tsinghua University, Beijing 100084, China

**Keywords:** Computer sciences, Environmental sciences, Earth sciences

## Abstract

Neighborhood physical disorder (PD), characterized by disruptions and irregularities in spatial elements, is associated with negative economic, social, and public health outcomes. Here, we present a protocol to quantitatively assess PD utilizing a range of metrics. We describe steps for collecting street views, constructing detection models using the YOLOv8 deep learning model, calculating PD scores, and quantifying changes in PD across streets and cites. This protocol serves as a methodological foundation for assessing PD in different countries and regions.

For complete details on the use and execution of this protocol, please refer to Chen et al.^1^

## Highlights

• Construct a dataset with commercial and customized street view images over time
• Implement deep learning YOLOv8 model to standardize disorder calculations
• Calculate disorder scores at city, street, and specific location granularity

## Abstract (editor highlights)

Publisher’s note: Undertaking any experimental protocol requires adherence to local institutional guidelines for laboratory safety and ethics.

## Graphical abstract

**Figure:**
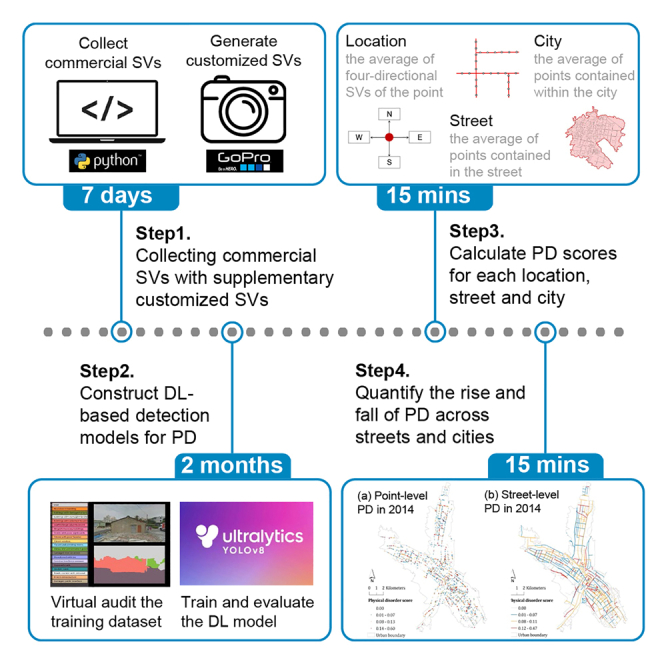

## Before you begin

### Method scope

The term “physical disorder” (PD), sometimes referred to as “neighborhood physical disorder” in urban settings, was initially introduced in 1990 by Skogan.^2^ This concept encompasses observable or perceptible visual indicators that disrupt the lives of residents and the quality of public spaces. These indicators include, but are not limited to, boarded-up or abandoned buildings, damaged and poorly maintained façades, vacant properties, inadequate signage, and even seemingly minor details like discarded cigarette butts. The negative consequences of PD are associated with economic performance, public health, and social stability, such as property depreciation, mental stress, fear, and crime. Empirical evidence strongly suggests that PD serves as an outward manifestation of urban decay.^2^^,^^3^ Neighborhood disorder can contribute to further environmental deterioration, resident migration, neighborhood instability, and ultimately, an increase in crime rates and community decline.^4^^,^^5^ Moreover, studies in public health and sociology have investigated the negative association between neighborhood disorder and individual health. Neighborhood disorder has been linked to detrimental behaviors and health outcomes such as overeating, reduced physical activity, alcohol abuse, and obesity.^6^^,^^7^^,^^8^^,^^9^ And living in a disorderly environment has been suggested to cause stress, fear, anxiety, and distrust among residents.^3^^,^^6^^,^^10^ It can even lead to a higher risk of recurrence among patients discharged after acute myocardial infarction.^11^ A limited but growing body of literature has examined the issue of PD in urban spaces, particularly in identifying PD at a fine scale. However, several challenges exist. Firstly, the conceptualization and spatial representation of PD are unclear. Secondly, there is currently no effective and replicable method to measure PD at large-scale but with low-cost. Lastly, the spatiotemporal changes of PD are not well understood due to the lack of available data.

This protocol proposes a method that utilizes a vast amount of street view images, supplemented with self-captured street views, as input data for training. It employs deep learning models to quantitatively measure the PD of urban street spaces. The protocol consists of four main steps: data acquisition of street view images, construction of deep learning-based recognition models, computation of PD at various scales, and analysis of the temporal changes in PD. The protocol was implemented in Xining City, China, with the aim of demonstrating its effectiveness and efficiency in addressing the intended objectives.

### Define PD metrics

1. Drawing from traditional built environment auditing methods in existing urban research, an audit checklist for PD was developed. Through a comprehensive review of published literature on PD in urban studies, major categories representing different spatial features were identified.
2. Some indicators were derived from on-site surveys conducted, combined with feedback from pre-experiments, reflecting specific spatial characteristics, such as unauthorized construction/temporary structures, disorderly street vending, and unpaved roads.
3. Other indicators were drawn from well-established studies on PD, including those related to vacant and abandoned buildings, vacant and for-sale storefronts, abandoned vehicles, and illegal dumping, as referenced from the Project on Human Development in Chicago Neighborhoods (PHDCN), Pedestrian Environment Data Scan (PEDS), and the Irvine Minnesota Inventory.
4. Ultimately, a total of 23 secondary evaluation indicators were identified as constituting elements of PD (refer to Table 1). These elements will contribute to the research and development of a unified standard PD evaluation system.

**Table 1:** The elements of PD covered in previous studies

Category	PD elements	Sources
Buildings	Boarded up or abandoned buildings	Quinn et al.^12^
	Building with damaged facades	Grubesic et al.^5^
	Buildings with unkempt facades	Grubesic et al.^5^
	Graffiti, or evidence of graffiti on buildings, signs, or walls	Hoeben, Steenbeek and Pauwels^13^
	Illegal and temporary buildings	On-site survey
	Broken or boarded-up windows	Frye et al.^14^
Street businesses	Presence of entirely vacant buildings	Marco et al.^15^
	Stores with poor signboard	On-site survey
	Stores with poor facade	On-site survey
	Abandoned cars or bicycles	Mooney et al.^16^
Sanitation and greenery	Garbage, litter, or broken glass	Bader et al.^17^
	Empty bottles or cans	Mooney et al.^16^
	Cigarette butts	Brownson et al.^18^
	Poorly maintained landscapes	Sampson and Raudenbush^19^
	Condoms, needles or syringes and drug paraphernalia	Molnar et al.^20^
	Too many dogs, dog excrement on the street	Jackson et al.,^21^ Ruijsbroek et al.^22^
	Rodents	Musa et al.^23^
Roads	Street disrepair	Allen^24^
	Roads stacked with personal belongings	On-site survey
Infrastructure	Deteriorated recreational infrastructure	Sampson and Raudenbush^19^
	Broken infrastructure, exposed power lines	On-site survey
	Damaged public interface	On-site survey
	Construction fence remnant	On-site survey
5. In the context of our experiments in China, we adapted this framework by selecting indicators that align with the Chinese urban landscape. The selection process was conducted as follows:
  a. Seven auditors with professional backgrounds in architecture or urban planning carried out field surveys in randomly chosen streets in Beijing.
  b. They also conducted virtual audits using 1000 randomly selected Tencent Street View Images (SVIs) from the vast dataset of 4,876,952 SVIs encompassing 264 cities. The purpose of this comparative approach was to assess the concordance between the indicators in our checklist and what was observed both in the SVIs and during the field surveys.
  c. Indicators that were not identified in either the SVIs or the field surveys, such as empty bottles and cigarette butts, were subsequently excluded from the checklist. Conversely, factors that were commonly observed in the sample SVIs or during the field surveys, aligning with the definition of PD, were included in the list. These additions encompassed elements like illegal or temporary buildings, street vendors, and unpaved roads.
  d. Through this iterative refinement process, we arrived at a finalized list comprising five categories and 15 detailed factors. These factors collectively represent the indicators of PD within Chinese urban streets.

### State-of-the-art (SOTA) methods

The elements of spatial disorder are diverse and multifaceted, necessitating a variety of sophisticated quantitative methods for their measurement, including Administrative or Commercial Data Source Method (ACDS method), Field Survey and Questionnaire Method (FSQ method), Systematic Social Observation Method (SSO method) and Virtual Audit Method (VA method), all of which have advantages and disadvantages compared to the proposed method in the protocol (refer to Table 2).

**Table 2:** Main advantages and disadvantages of SOTA method

Method		Spatial scope	Spatial resolution	Temporal resolution	Cost	Time	Sources
Administrative or Commercial Data Source Method (ACDS method)		Depends on data sources	Depends on data sources	Annually	Low	Low	Wheeler^25^
Field Survey and Questionnaire Method (FSQ method)		Region or city scale	Study sites	Annually	High	High	Latkin^26^
Systematic Social Observation Method (SSO method)		Region or city scale	Sampling site	Annually	High	High	Sampson and Raudenbush^19^
Virtual Audit Method (VA method)	Remote sensing	City or global scale	Full coverage	Daily	Low	Low	Patino^27^
	UAV images	Region or city scale	Full coverage	Daily or monthly	High	High	Grubesic^5^
	Street views	City or Country scale	Full coverage	Annually	Low	Low	Bader,^17^Quinn,^12^Mooney^16^
Deep learning method (our method)		City or Country scale	Full coverage	Daily or monthly	Low	Low	Chen et al.^1^

### Hardware

A minimum local-memory of 8 GB required. A workstation with Graphics Processing Unit (GPU) is recommended to accelerate image processing speed.

For customized data collection, each agent must be equipped with a minimum of one GoPro 9 camera, two batteries (each with a 97-min battery life), a car window mount, and a 128 GB storage card (capable of storing 3 h and 50 min of 1080 K video at 24 frames per second). In scenarios requiring concurrent data collection from multiple vehicles or bidirectional capture, it is advisable to have multiple cameras on hand.

For more sophisticated data collection requirements, consideration can be given to newer GoPro versions like GoPro 12. These updated models typically boast higher resolution, increased frame rates, and advanced image stabilization technology, delivering superior-quality video data. (Source: https://community.gopro.com/s/article/gopro-camera-battery-life?language=en_US, accessed on October 8, 2023.)

### Software

#### Footpath mobile application and online software Roboflow

6. The protocol mandates the installation of the Footpath mobile application on a smartphone for route planning and navigation purposes.
7. Access to and utilization of Roboflow’s online software services for training data preparation can be achieved after registering for a free account.

### Anaconda python platform

A simplified strategy to run Python on any operating systems is to use Anaconda. Anaconda provides a number of Python data science packages which are suitable for developing machine learning and deep learning models. It can be easily installed on any operating system (OS) such as Windows, Linux, and macOS. It is recommended to use Python 3.x version as Python 2 is no longer maintained. This study utilizes Anaconda on the Windows 10 OS, which comes with Python 3.9 pre-installed.

8. Anaconda can be downloaded from https://www.anaconda.com/products/individual according to individual computer specifications.
9. Once Anaconda is installed, open up the Anaconda Navigator to launch the PowerShell Prompt console.

### Install CUDA and cuDNN on Windows

NVIDIA CUDA Deep Neural Network (cuDNN) is a GPU-accelerated library of primitives for deep neural networks. It provides highly tuned implementations of routines arising frequently in DNN applications.

10. Install up-to-date NVIDIA graphics drivers on your Windows system, downloaded from http://www.nvidia.com/Download/index.aspx?lang=en-us. The installed version of the NVIDIA driver can be viewed from the system information in the NVIDIA Control Panel. This study utilizes version 471.41 of the NVIDIA driver.
11. Install the CUDA Toolkit for Windows by downloading the corresponding version of CUDA from https://developer.nvidia.com/cuda-downloads and subsequently executing the downloaded executable. Please verify the compatibility at the following link: https://docs.nvidia.com/deploy/cuda-compatibility/index.html#binary-compatibility__table-toolkit-driver*.* For the driver version 471.41, the corresponding CUDA version is 11.4.
12. Downloading cuDNN for Windows from https://developer.nvidia.com/rdp/cudnn-download.
13. Unzip the cuDNN package, copy the following files from the unzipped package into the NVIDIA cuDNN directory.

### Ultralytics YOLOv8 model

YOLOv8 is the latest family of You Only Look Once (YOLO) based Object Detection models from Ultralytics providing state-of-the-art performance. The object detection of PD factors is realized using Ultralytics YOLOv8. Install YOLOv8 via the ultralytics pip package for the latest stable release.

> pip install ultralytics

> conda install pytorch torchvision torchaudio cudatoolkit=11.4 -c pytorch

### QGIS software

QGIS (Quantum GIS) is an open-source Geographic Information System software used for processing road networks, PD data management, analysis, and visualization. Below are the steps to install QGIS 3.20.0 on the Windows OS.

14. Visit the official QGIS website: https://www.qgis.org/, select the version that matches your operating system, and download it.
15. Double-click the downloaded installer to launch the installation wizard.
16. Complete the installation and launch QGIS to start using it.

## Key resources table

**Table:** 

REAGENT or RESOURCE	SOURCE	IDENTIFIER
**Deposited data**		

Urban boundary	Own calculations	N/A
Street network	OpenStreetMap	https://www.openstreetmap.org/
Street views	Baidu	https://quanjing.baidu.com/

**Software and algorithms**		

Deep learning framework	PyTorch	https://pytorch.org/
Deep learning model	Ultralytics YOLOv8	https://github.com/ultralytics
Annotation tool	Roboflow	https://roboflow.com/
Spatial analysis tool	QGIS	https://www.qgis.org/

**Other**		

Dedicated vehicle	Taxi	N/A
Cameras with brackets	GoPro 9	https://gopro.com/en/us/shop/cameras/hero9-black/CHDHX-901-master.html

## Step-by-step method details

The actual time required for each computational step will largely depend on the scale of the studied city and the quantity of street views involved. The more city streets to analyze, the longer the time required. It is important to note that street view data can be purchased from several commercial companies through Application Processing Interfaces (APIs). Google Street View covers 100 countries across North and South America, Europe, and Oceania. In countries where it doesn’t provide coverage, alternative street view services are available. For instance, in China, Baidu currently conducts street view collection and continues to update, while Tencent Street View ceased updates and services in 2015. For areas or time periods not covered by commercial street views, a self-organized data collection method can be employed. The time frame for data acquisition will be determined by the time taken to scan specific areas using a vehicle. This article uses the city of Xining in China as an example to illustrate the time intervals for each step, including acquiring commercial street views and supplementing with self-collected data.

### Collecting commercial SVs with supplementary customized SVs

**Timing: 2 days (1 s per image, 42,828 images in total); 5 days (40 km/h, 1,440 km)**

The objective of this step is to acquire SVs within the scope of research to facilitate the subsequent identification of the elements of PD in the SVs. Initially, commercial SVs are collected from the Baidu Maps. For years or areas lacking commercial SV services, one may prepare personal recording equipment to capture and supplement data according to pre-designed routes.

Resource levels: [Intel Core i7-8750H/16 GB RAM/ 64 bit Windows 10/NVIDIA GeForce RTX 2070].

**Figure 1:**
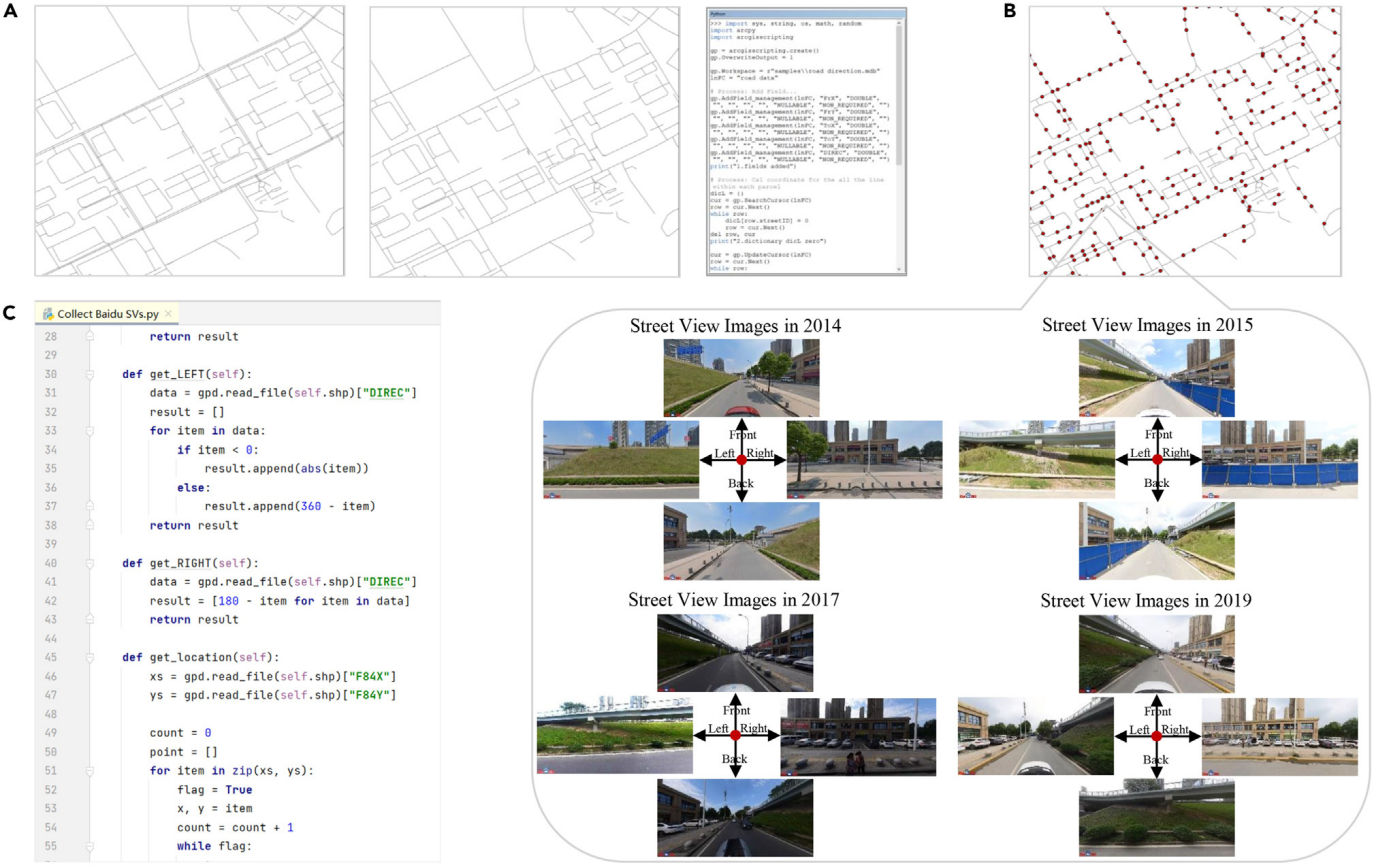
A commercial SVs collection workflow The workflow consists of three main steps: (A) simplifying roads and calculating road directions, (B) generating sampling points at certain distance intervals, and (C) obtaining multi-year street view images of each sampling point from Baidu Maps.

**Figure 2:**
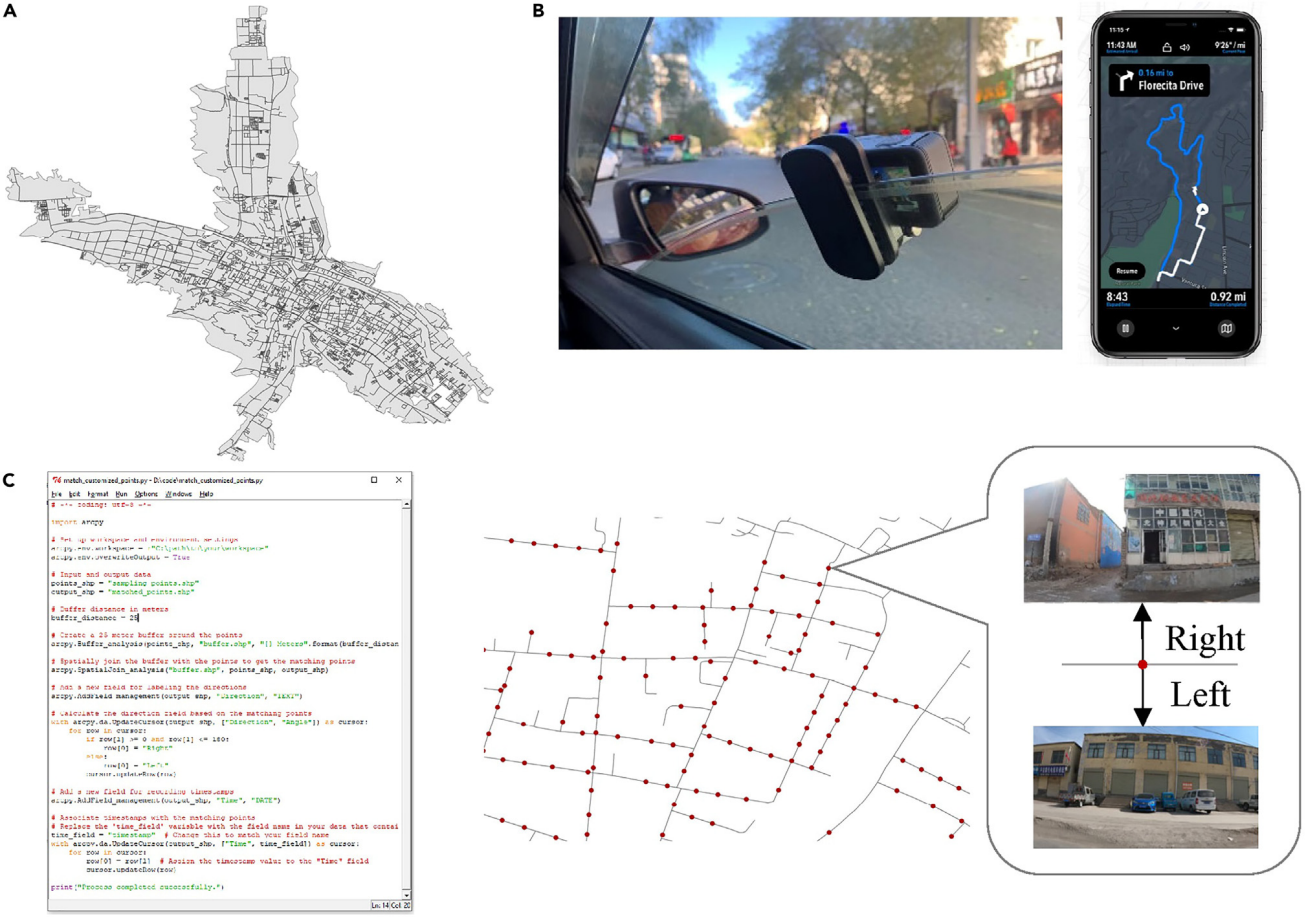
A customized data collection and data post-processing workflow The workflow consists of three main steps: (A) determining the research area and obtaining routes, (B) conducting data collection using a GoPro camera and navigation application Footpath, and (C) performing data post-processing using ArcPy to match the street view points with GPS points.

**Figure 3:**
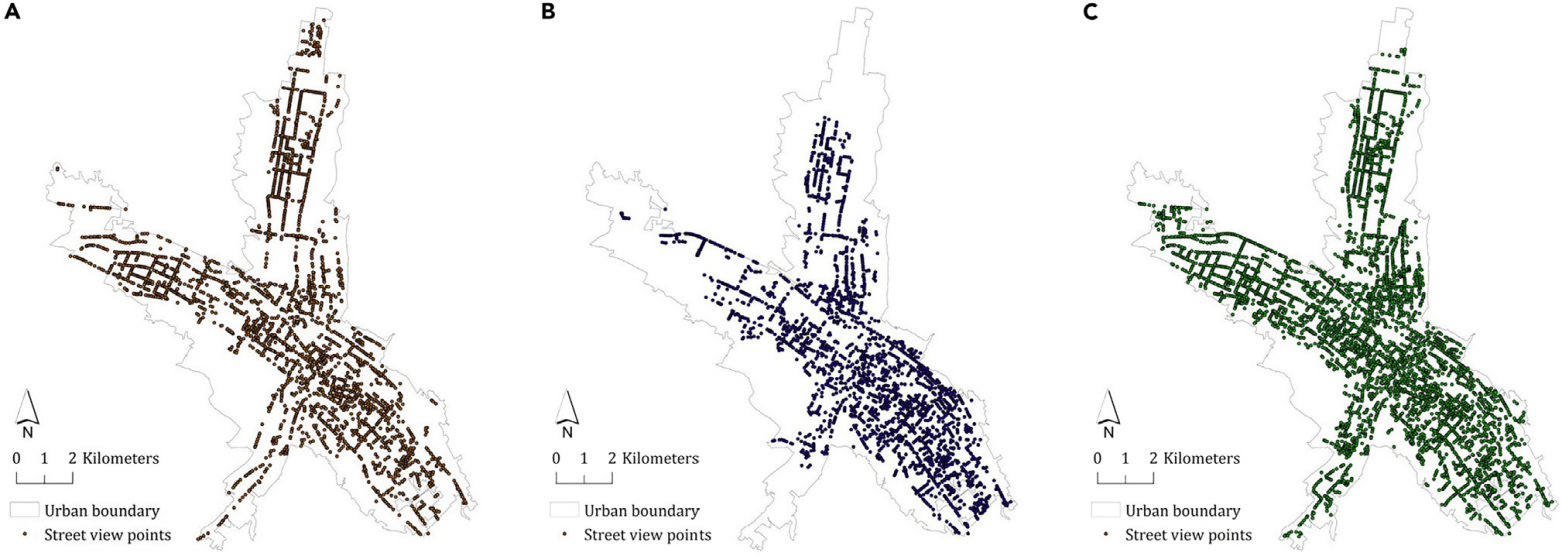
Contextual of spatial-temporal street view data (A) 2014 Baidu street views: 2,766 points capturing four directions (front, left, back, right). (B) 2018 Baidu street views: 2,434 points capturing four directions. (C) 2022 customized street views: 4,573 points capturing left and right directions.

1. Collect commercial SVs.
  a. Download the road data covering the research area for free from the Open Street Map website (https://www.openstreetmap.org/).
  b. Use the “Clip” tool in QGIS 3.20.0 to accurately clip the road data based on the urban boundary.
  c. Use the “Split Line at Vertices” tool in QGIS 3.20.0 to separate all roads at their vertices.
    i. Create a new field in the road data named "id" (set the field type as short integer).
    ii. Assign a value of 1 to the "id" of all roads.
  d. Use the "Merge Divided Roads" tool, with the merge field set to "idd."
    i. Adjust the merge distance within the range of 2–20 m to simplify the roads.
    ii. Repeat the process several times to achieve road simplification.
  e. Open the Python Integrated Development Environment (IDE) and access the "Road Direction.py" script.
    i. Set the path of the folder saving the code and the road data (filePath = r''samples' '), the name of the road data (data = gpd.read_file(path.join(filePath, 'road.shp'))).
    ii. Specify that the name of the output result is "road_direction.shp" (data.to_file(path.join(filePath, ' road_direction.shp'))).
  f. Run the code, which will add the coordinates of the starting point of each road and the "DIREC" field indicating the direction of the road to the road data.
  g. Access the Road Direction.py script.
    i. Set the path to the folder where the previous calculation results are saved (filePath = r’samples'), the name of the previous calculation results (line_layer = gpd.read_file(path.join(filePath, 'road_direction.shp'))).
    ii. Specify the name of the output result as sampling_points.shp"(point_layer.to_ file(path.join(filePath, 'sampling_points.shp'))).
    iii. Adjust the distance parameter to generate a sampling point every 50 m (distance + = 50).
  h. Run the code to calculate the road direction.
    ***Note:*** The output is sampling points of SVs, including the ID, direction, and latitude and longitude coordinates of each point.
  i. Apply for an API Key (AK) for coordinate conversion in Baidu Map Open Platform (https://lbsyun.baidu.com/) to get a code composed of numbers and letters.
  j. Open the Python Integrated Development Environment (IDE) and access the "Collect Baidu SVs.py" script.
    i. Adjust the image focus to 85 (fovy = 85).
    ii. Set the image size to 800∗400 (width = 800& height = 400).
    iii. Set the image pitch to 0 (pitch = 0) in the code. Enter the API Key (ak = sample).
    iv. Set the path of the folder saving the code (workSpace = r’samples'), the path of the sampling point data ( shp = r’samples\sampling_points.shp').
    v. Specify the output image folder location(save_path = r’samples\SVs').
  k. Run the code to obtain the SVs.
    ***Note:*** The SVs are saved in the specified folder and named "year_pointID_direction." Each point corresponds to images of four different directions, such as "2016_550_F.JPG," "2016_550_B.JPG," "2016_550_L.JPG," and "2016_550_R.JPG." It should be noted that not all sampling points have available SVs. When no SV is available for a sampling point, the output will be "There is no street view available at this location."
    Figure 1.
2. Generate customized SVs in the absence of commercial SVs.
  a. Firstly, identify the streets where street view images are not available.
  b. Employ QGIS’s toolbox "Network Analysis Library" using the shortest path tool to generate the shortest paths covering these streets.
  c. Save the resulting path file in .kml format for importing into Footpath mobile application that supports navigation usage.
  d. During the data collection process, equip yourself with a GoPro 9 camera and a 3 W mAh rechargeable power bank that can support 10 h of continuous data collection throughout the day.
    ***Note:*** For optimal results, mount the GoPro camera on a bracket fixed to the right window to capture one side of the road or on both the left and right windows to capture both sides simultaneously. This dual-camera setup is particularly beneficial for narrower streets, allowing you to record both sides in a single pass. However, for broader avenues, it's advisable to traverse them twice to ensure comprehensive data capture, without obstructions from moving vehicles and medians. The camera should maintain a 5° tilt angle, primarily facing the commercial areas along the street. Ensure that the videos are recorded in video mode with a standard view setting of 1920 × 1080 for clear and detailed footage.
  e. Launch the Footpath mobile application to initiate navigation and simultaneously record the GPS route.
    ***Note:*** The collected dataset includes GPS-recorded routes (.gpx) and video files (.avi) capturing the street view. Due to the 12-min limit per GoPro video, the videos are segmented into multiple files.
  f. Match the sampling points with GPS points by first calculating the direction for each point in the GPS file based on GPS tracks.
  g. Using the points from the "sampling points.shp," search for GPS points within a 25-meter buffer zone. If the direction is consistent, label it as "right"; if the direction is opposite, label it as "left."
  h. Simultaneously, associate the timestamp of the discovered GPS points with the "sampling points.shp" file and record it as "time."
    ***Note:*** Please refer to the code "Match_GPS_and_Sampling.py" for more details. The Python package used for this purpose is Shapely.
  i. Using the timestamps from the "sampling points.shp" file, retrieve the corresponding video frames from the video at the respective times and name the frames as "collection year_pointID _direction.jpg."
    ***Note:*** Please refer to the code "Retreive_frame.py." The Python package used for this is cv2 (OpenCV).
    Figures 2 and 3.

### Construct DL-based detection models for PD

**Timing: 2 months**

The purpose of this step is to establish a robust indicator system for evaluating PD in Chinese cities and to develop a deep learning model based on this system.

Resource levels: [Intel Core i7-8750H/16 GB RAM/ 64 bit Windows 10/NVIDIA GeForce RTX 2070].

3. Determine the virtual criteria for PD factors.
  a. In accordance with prior research, a comprehensive checklist comprising 15 common PD factors in five distinct categories is constructed. This checklist serves as a foundational framework for assessing the presence and extent of PD.
  b. Ensure a standardized approach to conducting virtual audits.

***Note:*** Please refer to the "*Virtual Audit Manual for Physical Disorder.pdf*" for the virtual audit standards pertaining to these factors. If region-specific appearances or characteristics exist, it is essential to incorporate them into the manual, thereby ensuring a unified standard across all auditors involved in the dataset labeling process. Each factor is supplemented with a sample image that effectively illustrates its manifestation and facilitates its identification within the Street View Images (SVIs).

4. Virtual audit the training dataset.
  a. Sign up and log on to the website of Roboflow (https://roboflow.com/).
  b. Add a workspace and select “academic.” Create a project and choose “object detection” as the project type. Set a project name.
  c. Click “upload” and select “select files” to upload the images you want to train on the website.
  d. After the import is complete, double-click on any image to annotate it.
    i. Use the rectangle tool to draw bounding boxes around the objects and name the object category in the pop-up window.
    ii. Once all the images are annotated, click “save and continue.”
    iii. Select “Split Images Between Train/Valid/Test” and automatically divide the dataset into training, validation, and testing sets based on 80%, 10%, and 10% respectively.
  e. Click “health check” to review the class balance and ensure that all categories are well presented.
  f. For underrepresented classes, add additional training images and annotations.
  g. Click on "Generate" to export the dataset to local computer.
    i. Set up preprocessing steps, including "Filter Null," which requires 80% of the images to be annotated as not all images display PD factors.
    ii. Set "Tile" as 2 x 2 to facilitate the detection of small objects.
    iii. Configure augmentation steps, including horizontal flipping, 20% cropping, and 15 degrees of vertical and horizontal shearing, as this significantly enhances detection capabilities for objects at different angles.
  h. Click “export” and choose the “yolov8 format.” Export the compressed file to your computer. After decompressing, rename the folder as “pddata.”

**Table 3:** Model performance for PD factors

PD factors	Number of Images	Number of Instances	mAP@50 (%)
Abandoned buildings	598	2,272	94.1
Buildings with damaged facades	4,210	7,157	83.4
Buildings with unkempt facades	15,902	15,902	79.9
Graffiti/illegal advertisement	12,645	13,909	80.7
Illegal/temporary buildings	4,618	12,930	71.6
Stores with poor signboards	6,427	21,851	84.6
Stores with poor facades	2,843	10,803	89.8
Vacant and pending stores	2,736	7,387	90.3
Messy and unmaintained greening	7,726	13,134	81.8
Garbage/litter on street	5,096	6,115	82.4
Construction fence remnant	626	1,940	96.9
Broken roads	13,061	14,367	80.5
Roads stacked with personal belongings	1,074	1,181	97.6
Broken infrastructure	11,224	11,224	91.0
Damaged public interface	2,964	8,002	84.1
All classes			85.9

Figure 4.

5. Train and evaluate the deep learning model.
  a. Open the command prompt and navigate to the dataset environment.
    i. Enter the command:
      > yolo task=detect mode=train model=yolov8n.pt data=pddata/data.yaml epochs=200 imgsz=640 workers=1 batch=4”
    ii. Modify the suffix after "data = " to the path of generated dataset.
    iii. The learning rate, batch size and epochs are 0.001, 4 and 200, respectively.
      ***Note:*** After training is complete, the trained model will be saved in the current environment’s path, specifically in the “runs\detect\train\best.pt” folder.
  b. Based on the recommendations from the YOLOv8 official website, it is initially recommended to train using default parameters, with a focus on preparing a well-structured training dataset.
    ***Note:*** Here are some tips to improve the quality of training results:
    Diversity of images and instances: It is recommended to have at least 1,500 images and 10,000 labeled object instances per class to achieve optimal results. Ensure that the dataset represents the real-world deployment environment. Include images from different times of day, various seasons, diverse weather conditions, varying lighting conditions, different angles, and from different sources (such as web scraping, local collection, and different camera captures).
    Label consistency and accuracy: Accurate labeling of all instances for all classes in every image is essential. Avoid partial labeling, as it may lead to suboptimal results. Ensure that labels closely enclose each object. There should be no gaps between objects and their bounding boxes, and each object must have a corresponding label. Before training, review "train_batch∗.jpg" to validate the correctness of labels. This helps ensure accuracy and identify any labeling errors.
    Background images: Consider adding background images to the dataset. These images do not contain any objects and can help reduce false positives (FP). It is recommended to include around 0%–10% background images (e.g., COCO dataset has 1,000 background images, which constitute approximately 1% of the total dataset). Background images do not require labels.
    Following these recommendations can enhance the performance and accuracy of the trained model. The training results of this study are presented in Table 3. The accuracy of the model is represented by mAP@50 (%), which signifies the mean average precision (mAP) when the predicted bounding box overlaps with the true bounding box by at least 50%.

**Figure 4:**
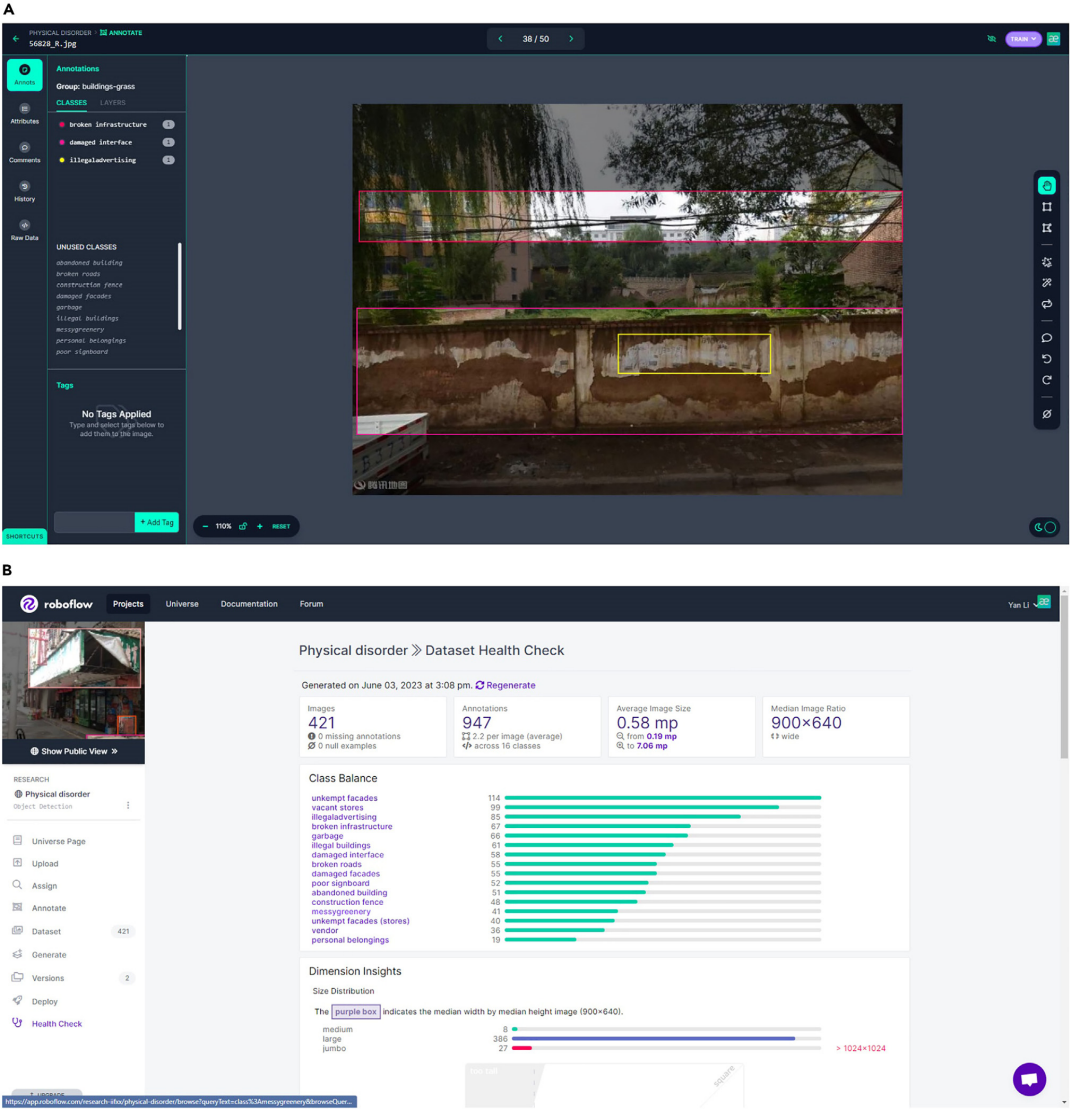
Preparation of the training dataset using Roboflow (A) Uploading images to Roboflow and annotating them using rectangular annotation tools; (B) Checking the quality of the training dataset using the Dataset Health Check tool, ensuring balanced and well-represented training samples indicated by the green color.

### Calculate PD scores for each location, street, and city

**Timing: 15 min (14 ms per image, 42,828 images in total)**

The purpose of this step is to aggregate PD scores from each street view image to each point, street, and city.

Resource levels: [Intel Core i7-8750H/16 GB RAM/ 64 bit Windows 10/NVIDIA GeForce RTX 2070].

6. Identify PD elements from each image.
  a. Open the Python Integrated Development Environment (IDE) and access the “Prediction_ yolov8.py” script.
  b. Specify the image location for computation and set the storage directory for the results.
  c. Click on the “Run” button to generate the predictions based on the specified settings.
    ***Note:*** The results consist of predicted images and annotation outcomes. The annotation outcomes include label categories and their corresponding bounding boxes, stored in text format.
  d. To review the prediction results, navigate to the target location where the images and labels are stored, and examine the generated predictions.
  e. Run Python script “Result_conversion.py” to manipulate the prediction results into CSV format, including the street view point ID and the existence of each PD element in each direction.
7. Calculate the PD score at each location.
  a. Open QGIS and load the street view point data. Join the CSV file containing the detection results with the street view points using the "Join" tool, based on the street view point ID.
  b. Export the associated layer and save it as a "pd_point.shp" shapefile.

***Note:*** Aggregate the results from different directions to that specific location. For each location’s PD value, $D_{point\_i}=\frac{\sum_{1}^{j}\sum_{1}^{k}p_{jk}}{4\times 15}$, where *D*_*point_i*_ is the disorder score of sampling point *i*. *p*_*jk*_ is the fraction of the *k*_*th*_ disorder element of the street image in direction *j*. *j* = 1, 2, 3, 4 refers to the north, south, west, and east directions, while *k* = 1–15 refers to the 15 disorder elements.

8. Calculate the PD score for each street.
  a. Open QGIS and load street data and "pd_point.shp."
  b. Access the “Join” tool from the street data layer, select the "pd_point.shp" as the layer to join to this layer, and calculate the average value of the fields for all data that fall within that street line. Save the joined data as “pd_street.shp.”

***Note:*** Aggregate the disorder values of all points on the street. For the disorder value of a particular street, follow the formula, $D_{street\_k}=\frac{\sum_{1}^{n}D_{point\_i}}{n}\left(i=1,...,n\right)$, where *D*_*street_k*_ is the disorder value of street *k*; *n* is the number of sampling points in street *k*.

9. Calculate the PD score for each city.
  a. Open QGIS and load urban boundary data and "pd_street.shp."
  b. Access the “Join” tool from the urban boundary data layer, select the "pd_street.shp" as the layer to join to this layer, and calculate the average value of the fields for all data that fall within that city boundary. Save the joined data as “pd_city.shp.”

***Note:*** Aggregate the disorder values of all streets within that city. For the disorder value of a city, follow the formula, $D_{city\_l}=\frac{\sum_{1}^{m}D_{point\_i}}{m}\left(i=1,...,m\right)$, where *D*_*city_l*_ is the disorder value of city *l*; and *m* is the number of sampling points in city *l*.

### Quantify the rise and fall of PD across streets and cities

**Timing: 15 min**

The purpose of this step is to calculate the changes of PD across different years for streets and cities.

Resource levels: [Intel Core i7-8750H/16 GB RAM/ 64 bit Windows 10/NVIDIA GeForce RTX 2070].

10. Based on the correspondence of streets across different years, the improvement and deterioration of PD can be determined.
  a. Deteriorating PD is identified when the PD value in the later year is higher than that in the previous year, while improvement occurs when the PD value in the later year is lower than that in the previous year.

***Note:*** Each acquired street and location comes with a unique identifier, which remains the same across years. These identifiers enable PD in different years to be matched, which is a great advantage over other state-of-art methods. Due to the varying city boundaries and streets updated annually, the comparative analysis over multiple years relies entirely on available data points from each respective year.

***Note:*** For each street, considering the varying length and number of street points on each street, the calculation involves determining the proportion of upgraded street points and the proportion of deteriorated points on that street. However, the raw ratio of PD improvement and deterioration is statistically too varied when the denominator is small, so streets with too few street view points (<10) are omitted.

## Expected outcomes

We use the information from over 300 cities in China in 2015 as an example to explain the calculated expected results.^1^ Based on the predictions from deep learning models, the study reveals the PD in the street networks of Chinese cities and uncovers significant patterns at both the street and city scales. At the street level, the PD distribution values of street spaces in Chinese cities exhibit a widespread presence of PD, although the spatial quality of most streets remains moderate. Analyzing the PD values of different categories reveals that high PD in terms of buildings, roads, and commercial areas is the primary manifestation of street PD in China. By combining other indicators of the built environment, including street length, functional diversity, and distance to the city center, the study uncovers the impact factors and mechanisms behind the PD of streets. At the city scale, spatial clustering of street PD reveals the spatial patterns of disorder in street spaces, including single-centered, linear concentration along major roads, and multi-centered distributions across cities.

We have also showcased the temporal changes in PD in Xining City, China, using street view data from multiple sources. The data used for this study included imagery from Baidu Street View, which encompassed the following time points: May 2014, August 2016, and June 2018, but have not been updated since then. Subsequently, we collected data for March 2022 and March 2023 from the mobile sensing platform through a self-organized effort. For consistency and to minimize potential seasonal variations in the landscape (such as foliage changes between autumn and winter), we specifically selected images from 2014, 2018, and 2022, ensuring a consistent time interval of four years between each time point. Therefore, the selected time scale for the Street View images in this study maintained a four-year interval between captures. This selection aimed to provide a coherent temporal framework for our analysis while taking into account seasonal variations.

**Figure 5:**
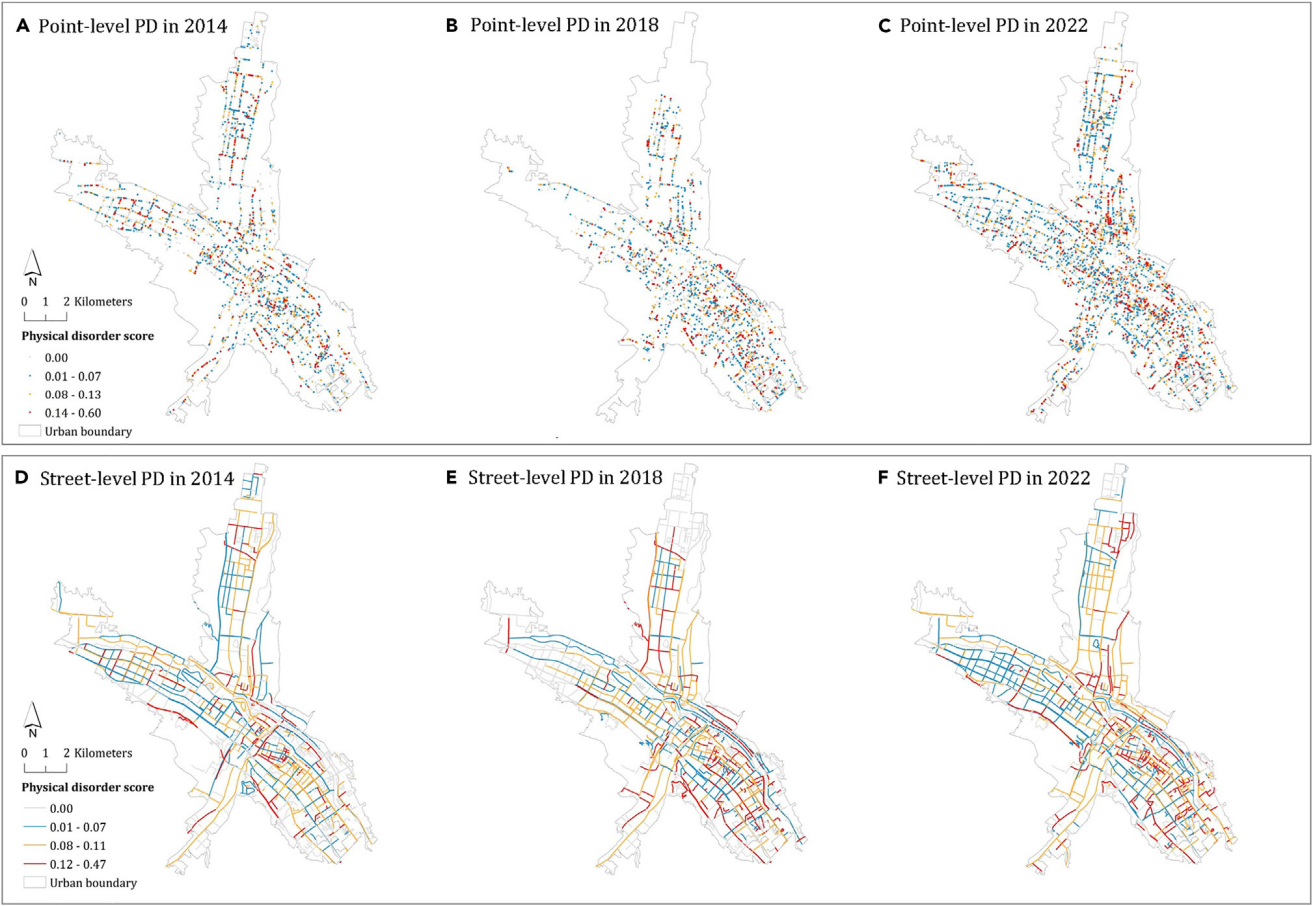
The evolution of PD at the point and street levels in Xining City, China, from 2018 to 2022 Subplots in (A–C) sequentially represent point-scale visualizations from 2014 to 2022, and subplots in (D–F) sequentially represent street-scale visualizations from 2014 to 2022. Data for the years 2014 and 2018 were sourced from Baidu’s commercial street view, while the data for 2022 were collected through self-collection efforts.

The visualization was performed at both the point and street levels in Figure 5. The average street PD values for the entire city in 2014, 2018, and 2022 were 0.04, 0.04, and 0.08, respectively, exhibiting an ascending trend over the years. This observation suggests an overall deterioration in spatial quality.

At the specific indicator level, a detailed analysis can be conducted. For example, the changes in abandoned buildings^28^ *c*an be revealed, thereby uncovering specific aspects of PD. Additionally, integrating with health outcomes can help explore the relationship between PD and health.

## Limitations

The current PD of images relies on binary indicators to identify PD has its limitations. For instance, the effect of a single abandoned building on the street would be different from that of a row of abandoned buildings. However, in this exploratory study, our focus was primarily on determining the presence of PD in urban street spaces rather than quantifying the level of disorder. Future plans could involve implementing the image segmentation model, which would not only allow us to identify the presence of PD factors but also provide information about the proportion of each factor’s area, such as the area of untidy facades or graffiti, as well as the number of vacant street vendors present. However, image segmentation models require annotating the shape of each object, resulting in significant more workload. Due to the lack of large-scale annotated databases, it still poses significant challenges.

## Troubleshooting

The most common issues arise in the preparation of training samples and the accuracy of model detection.

### Problem 1

How to decide the value of this distance parameter? (step 1g in step-by-step method details)

### Potential solution

The selection of a 50-meter distance for generating sampling points in the street view data collection phase was influenced by insights from historical literature, which have applied various resolutions for street view data acquisition. These resolutions have spanned from 20 m,^29^ 50 m^30^^,^^31^^,^^32^^,^^33^ to 100 m^34^^,^^35^(Helbich et al., 2019, Wang et al., 2019) to 25 miles.^36^ While choosing 20 m would have ensured coverage of all buildings on the street, we chose 50 m intervals for our analysis given the time required to acquire street view imagery and the efficacy of previous studies that have used 50 m as the standard for intervals, which is a trade-off between the cost of time and the storage space needed to consider when downloading data for further research into national-scale analyses. It can also be adjusted according to different research purposes.

### Problem 2

The API Key of Baidu Maps Open Platform has a daily usage quota, which can impede the pace of data collection. Moreover, Baidu Maps might impose access restrictions, especially when collecting Street Views (SVs). Frequent access can lead to IP restrictions, further affecting the data collection process (step 1i in step-by-step method details).

### Potential solution

A paid or enterprise version of API Key can be applied to increase the daily limit (https://lbsyun.baidu.com/, accessed August 22, 2023). Or multiple agents can work in parallel to access images through multiple API keys. Another strategy is to deploy multiple agents to work simultaneously, accessing images using different API keys. However, when faced with IP access restrictions due to high-frequency access, the most practical approach is to pause the data collection and resume once the IP access restriction is lifted.

### Problem 3

There are commercial SVs that are taken in tunnels or in scenes with very low brightness and do not reflect PD (step 1k in step-by-step method details).

### Potential solution

A quick method is to judge based on the size of the image. These SVs are affected by light and have large black areas, and they are smaller than SVs collected on normal roads. The size of a normal SV is between 150-400 kb, while the size of a SV taken in a tunnel or scene with extremely low brightness is below 100 kb. The images that cannot be recognized properly can be excluded by inspecting the smaller images.

### Problem 4

There are instances where Baidu Street View images cannot be opened after downloading (step 1k in step-by-step method details).

### Potential solution

For locations where the downloaded image files have a file size of 0, it is recommended to retry downloading those locations again.

### Problem 5

What precautions should be taken when conducting data collection independently? (step 2d in step-by-step method details).

### Potential solution

Consider the weather conditions during data collection and avoid rainy, snowy, foggy, or windy weather to ensure clear and sharp lens captures that accurately depict the urban environment.

### Problem 6

How can the PD indicators be expanded (step 3 in step-by-step method details)?

### Potential solution

To expand the PD indicators, it is recommended to augment the training sample data with new indicators. Upload the augmented data to Roboflow and export the updated training dataset. Then, proceed with retraining the model using the augmented dataset. Once the new object detection model is obtained, it can be used to detect the PD elements.

### Problem 7

How to determine the number of training samples? (step 4c in step-by-step method details)

### Potential solution

In the context of training the YOLOv8 model, the guideline typically recommends preparing a dataset of at least 1500 training images (https://docs.ultralytics.com/yolov5/tutorials/tips_for_best_training_results/#training-settings, accessed on September 25, 2023). Therefore, to optimize the training results, it is recommended to have a substantial number of images per class, with a minimum of 1500 images per class. Additionally, for each class, it is beneficial to include at least 10,000 instances of labeled objects. To ensure a dataset representative of real-world scenarios, it is essential to incorporate image variety, including different times of day, various seasons, diverse weather conditions, varying lighting situations, multiple angles, and images from different sources, such as those scraped online, collected locally, or captured with different cameras. Following these guidelines will enhance the training process and lead to more robust and accurate models. However, it’s important to note that this is a general recommendation, and there can be some flexibility in practice.

Additionally, when using Roboflow, if there is a shortage of training samples, it will prompt "underrepresented." In such cases, you can supplement the samples until they are "well represented." During the training process, it is advisable to closely monitor the training curves and performance metrics. Monitoring the loss function during the training process is typically visualized in the form of charts. A continuous decrease in the loss function that tends towards stability is generally a positive indicator. When the accuracy reaches your desired level, it can be considered that the model has achieved satisfactory performance. Additionally, observing the loss and accuracy on a validation set is essential to ensure that the model does not overfit, meaning it performs well on the training data but poorly on new, unseen data.

The key consideration here is the quality and representativeness of the training dataset. A smaller dataset that is diverse and effectively captures the relevant features of the urban environment may still yield satisfactory results. The specific number of images required may vary depending on the complexity of the task, the diversity of the scenes, and the quality of the training data.

### Problem 8

Do we need to manually annotate each image using rectangular annotation tools? (step 4 in step-by-step method details)

### Potential solution

In the context of our study, we have employed an object detection model, necessitating the utilization of annotation tools to delineate the rectangular shape corresponding to each disorder element within the images. However, for the 15 distinct categories of spatial disorder elements, there is no requirement for readers to manually annotate an entirely new training dataset. We have already meticulously annotated the entire dataset and subsequently provided a pre-trained model for the convenience of our readers.

In addition, different deep learning methods require different annotation techniques. We meticulously compared three cutting-edge models based on image classification (MobileNet V3, developed by Howard et al.^37^), image detection (YOLOv8, developed in our research), and image segmentation (SegNet, proposed by Badrinarayanan et al.^38^). The complexity of annotations for these models ranges from simple categorization to drawing bounding boxes and outlining object contours.

### Problem 9

Why is the accuracy on my images not as high as reported by the model? (step 5b in step-by-step method details)

### Potential solution

First, check if the viewpoint of the detection images aligns with the viewpoint of the training images. If they are not consistent, you need to add additional training samples from your own images that represent the features present. Then, for cases where the accuracy is not high for a specific category, you can check the annotated samples and make sure that the samples contain salient features. By doing this, the model can benefit from a larger and more diverse training dataset, which is likely to improve the detection performance for the objects that were previously missed.

## Resource availability

### Lead contact

Further information and requests should be directed to the lead contact, Ying Long (ylong@tsinghua.edu.cn).

### Materials availability

This study did not generate new unique materials.

### Technical contact

Further technical queries should be directed to the techinical contact, Yan Li (yanli427@hotmail.com).

### Data and code availability

The code has been deposited to Mendeley Data: https://doi.org/10.17632/d3d4h5bvss.2. The citation for the associated dataset is as follows:

Li, Yan; Ma, Yue; Long, Ying (2023), “Protocol for Evaluating Neighborhood Physical Disorder Incorporating Multiple Street View Sources,” Mendeley Data, V2, doi: https://doi.org/10.17632/d3d4h5bvss.2.

Any additional information required to reanalyze the data reported in this paper is available from the lead contact upon request.
